# Evaluation of drug-drug interaction between nacubactam and β-lactam antibiotic (cefepime, aztreonam, meropenem, or piperacillin) in Japanese healthy participants

**DOI:** 10.1128/aac.00282-26

**Published:** 2026-06-09

**Authors:** Jun Morita, Hiroki Sato, Masayo Sumiya, Risako Takaya, Kazuya Ishiwata, Shogo Matsumoto, Takuma Yonemura, Yuji Kumagai

**Affiliations:** 1 R＆D Division, Meiji Seika Pharma Co., Ltd. 13418 , Tokyo, Japan; 2 Souseikai Sumida Hospital 684790 , Tokyo, Japan; 3 Kitasato University Kitasato Institute Hospital 12877, Tokyo, Japan; Providence Portland Medical Center, Portland, Oregon, USA

**Keywords:** nacubactam, beta-lactamase inhibitor, clinical trial, phase 1, pharmacokinetics, safety cefepime, aztreonam, meropenem, piperacillin

## Abstract

We conducted an open-label study to assess the pharmacokinetics, safety, and tolerability of nacubactam—a β-lactamase inhibitor—administered alone or in combination with β-lactam antibiotic. Japanese healthy male participants received a single dose of nacubactam 2 g on Day 1, a single dose of β-lactam antibiotic (cefepime 2 g, aztreonam 2 g, meropenem 2 g, and piperacillin 4 g in Cohorts 1, 2, 3, and 4, respectively) on Day 3, and the combination of nacubactam and β-lactam antibiotic on Day 5. The study drugs were intravenously administered over 60 min. A total of 32 participants (8 in each cohort) were enrolled in the study. All participants completed the study and were included in the analysis. Exposures to nacubactam or β-lactam antibiotic (maximum plasma concentration [*C*_max_], area under the plasma concentration-time curve from time zero to the last quantifiable time [AUC_0−t_], and AUC from time zero to infinity [AUC_0−∞_]) were similar between the treatment periods (monotherapy vs combination therapy) in all cohorts. Coadministration of nacubactam and β-lactam antibiotic did not affect the pharmacokinetic profile of nacubactam or β-lactam antibiotic with the 90% confidence intervals for the geometric least squares mean ratios (combination therapy/monotherapy) of *C*_max_, AUC_0−t_, and AUC_0−∞_ contained within the equivalence range of 0.80 to 1.25 for all study drugs. Nacubactam and β-lactam antibiotics were well tolerated. All treatment emergent adverse events were mild in severity and resolved without treatment. Our results support the further clinical development of nacubactam coadministered with these β-lactam antibiotics.

## INTRODUCTION

Antimicrobial resistance is one of the greatest challenges to global public health, as stated by the World Health Organization (https://iris.who.int/server/api/core/bitstreams/0d3763c7-5559-473a-8403-f6f6774a0a5f/content). In particular, multidrug-resistant gram-negative bacteria have been considered a major health concern (1, 2). Thus, antibiotics that are active against these bacteria should be intensively developed (3). Considering that β-lactam antibiotics are the cornerstone for the treatment of bacterial infections and that the resistance in gram-negative bacteria is mostly mediated by β-lactamases, the combination of β-lactam antibiotic and β-lactamase inhibitor may be a promising option for the treatment of multidrug-resistant bacterial infections (4).

Nacubactam is a diazabicyclooctane developed by Meiji Seika Pharma Co., Ltd. (Tokyo, Japan). It strongly inhibits Class A and Class C β-lactamase and has direct antimicrobial activity by inhibiting penicillin binding protein 2 of several *Enterobacteriaceae* (5). Furthermore, it enhances the antimicrobial activity of β-lactam antibiotics (5). These favorable profiles led to the further development of nacubactam. In the non-clinical studies, nacubactam in combination with β-lactam antibiotic—such as cefepime, aztreonam, meropenem, or piperacillin—was active against gram-negative bacteria producing β-lactamase (6–10). In the single- and multiple-dose phase 1 studies conducted in Western countries, the pharmacokinetics (PK) of nacubactam following single (0.05 to 8 g) and multiple (1 to 4 g) doses was linear with the half-lives of 2 to 3 h (11). Nacubactam was well tolerated in these studies.

Recently, two randomized, placebo-controlled phase 1 studies were conducted in Japan (12). In these studies, the pharmacokinetic (PK) profile of nacubactam was similar to that obtained from the phase 1 studies in Western countries. No safety concerns specific to the Japanese population were observed. However, these placebo-controlled studies did not assess the drug-drug interaction in pharmacokinetics between nacubactam and β-lactam antibiotic. Thus, we conducted an open-label study to assess the pharmacokinetics, safety, and tolerability of nacubactam administered alone or in combination with cefepime, aztreonam, meropenem, or piperacillin in Japanese healthy adult male participants.

## RESULTS

### Participant disposition and baseline characteristics

A total of 32 Japanese male participants (8 in each cohort) were enrolled in the study. All participants completed the study and were included in the analysis. Baseline characteristics of participants were well balanced among the cohorts (Table 1). The mean age in each group ranged from 22.5 to 26.6 years. The mean weight in each group ranged from 59.68 to 62.91 kg.

**TABLE 1:** Baseline characteristics of the study participants

Characteristic^*a*^	Cohort 1 (*n* = 8)	Cohort 2 (*n* = 8)	Cohort 3 (*n* = 8)	Cohort 4 (*n* = 8)	Total (*n* = 32)
Age (year)					
Mean	24.6	23.0	22.5	26.6	24.2
SD	6.7	5.0	3.1	5.4	5.2
Height (cm)					
Mean	169.99	169.35	170.86	170.41	170.15
SD	2.49	6.75	6.04	6.42	5.44
Weight (kg)					
Mean	59.68	62.91	60.56	60.74	60.97
SD	6.91	6.11	6.77	9.91	7.29
BMI (kg/m^2^)					
Mean	20.56	21.96	20.64	20.76	20.98
SD	2.02	2.60	1.45	2.27	2.10
Serum creatinine (mg/dL)					
Mean	0.871	0.913	0.895	0.829	0.877
SD	0.077	0.098	0.078	0.083	0.084
Estimated glomerular filtration rate (mL/min)					
Mean	90.0	87.1	89.6	92.9	89.9
SD	7.6	9.3	7.8	9.3	8.5

^*a*^ SD, standard deviation; BMI, body mass index.

### Pharmacokinetics

**Fig 1:**
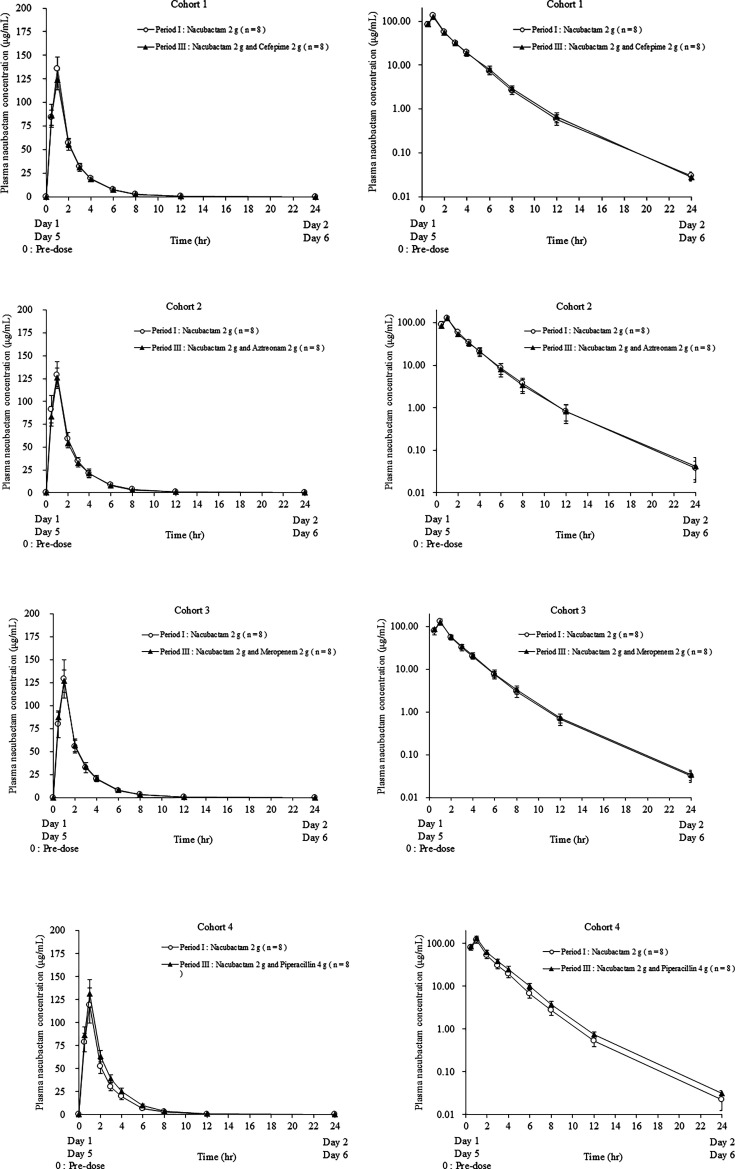
Mean plasma concentration-time profiles of nacubactam following a single infusion of 2 g over 60 min. Nacubactam was administered alone on Day 1 and in combination with β-lactam antibiotic on Day 5 in each cohort(linear and semi-log scale). β-lactam antibiotics administered in Cohorts 1, 2, 3, and 4 were cefepime (2 g), aztreonam (2 g), meropenem (2 g), and piperacillin (4 g), respectively.

**Fig 2:**
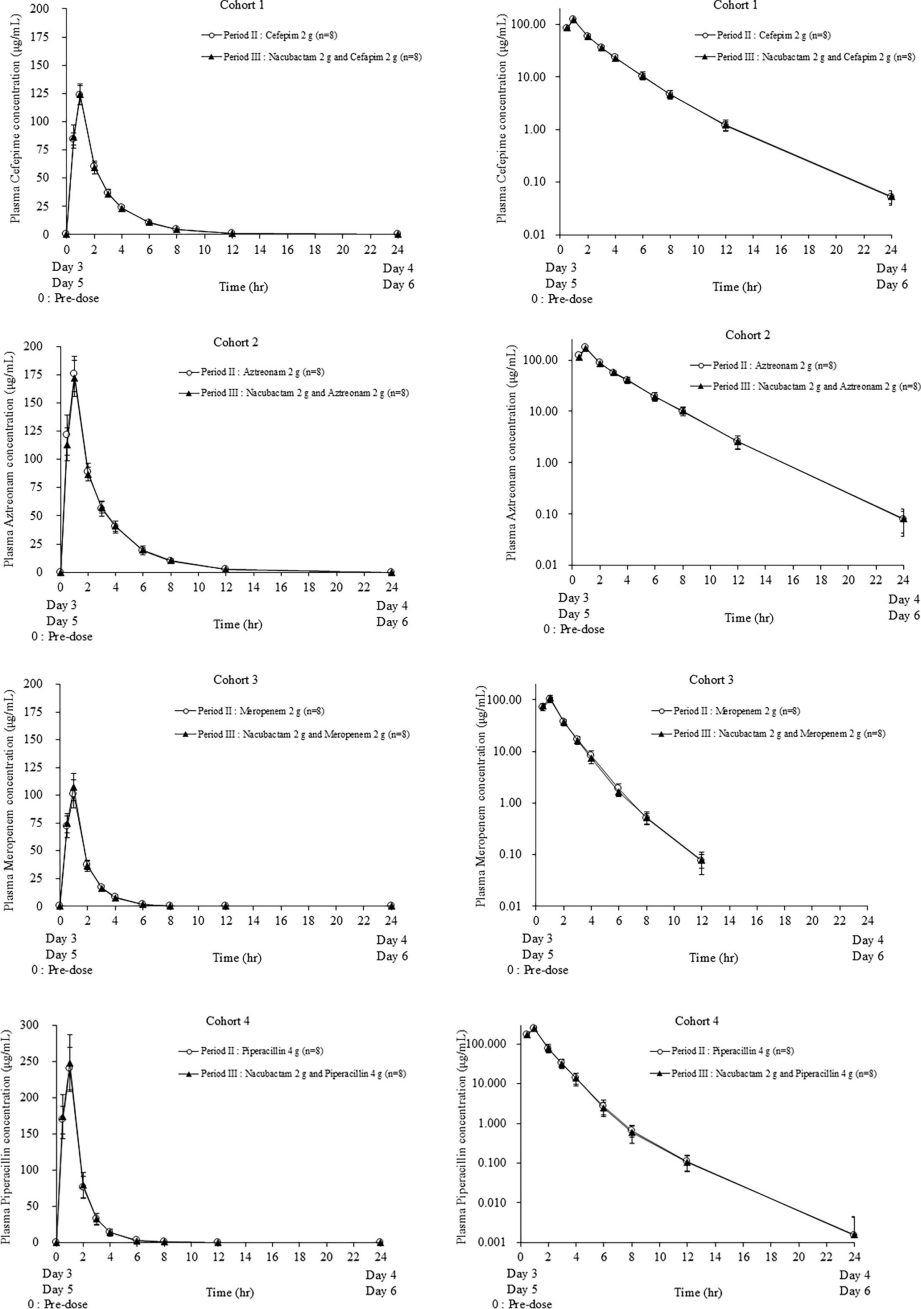
Mean plasma concentration-time profiles of β-lactam antibiotics following a single infusion over 60 min. β-lactam antibiotics (cefepime 2 g, aztreonam 2 g, meropenem 2 g, and piperacillin 4 g in Cohorts 1, 2, 3, and 4, respectively) were administered alone on Day 3 and in combination with nacubactam on Day 5 (linear and semi-log scale).

The plasma concentration-time profiles of nacubactam were similar between Periods I (administered alone) and III (coadministered with β-lactam antibiotic) in all cohorts (Fig. 1). Across the treatment periods and cohorts, plasma concentrations of nacubactam following a single infusion of 2 g peaked at the end of the infusion followed by a rapid decrease. The plasma concentration-time profiles of each β-lactam antibiotic (cefepime, aztreonam, meropenem, and piperacillin in Cohorts 1, 2, 3, and 4, respectively) were also similar between Periods II (administered alone) and III (coadministered with nacubactam) (Fig. 2). Whether administered alone or in combination with nacubactam, plasma concentrations of all β-lactam antibiotics peaked at the end of the infusion followed by a rapid decrease.

PK parameters for nacubactam were almost constant across the treatment periods and cohorts (Table 2). The mean maximum plasma concentration (*C*_max_) ranged from 118.6 to 135.9 µg/mL. The mean area under the plasma concentration-time curve from time zero to the last quantifiable time (AUC_0−t_) ranged from 266.3 to 319.4 µg∙h/mL. The mean AUC from time zero to infinity (AUC_0−∞_) ranged from 266.5 to 319.5 µg∙h/mL. The mean elimination half-life (*t*_1/2_) ranged from 2.3 to 2.5 h. Cumulative percentages of nacubactam excreted in urine within 24 h post-dose were approximately 90% irrespective of the treatment periods and cohorts. The mean *C*_max_, AUC_0−t_, and AUC_0−∞_ of the metabolites (M1 [open ring analog] and M2 [deaminated ethoxy analog]) were far lower than those of nacubactam (Table 3). PK parameters for each β-lactam antibiotic were similar between Periods II and III (Table 4). Cumulative percentages of each β-lactam antibiotic excreted in urine were also similar between the treatment periods.

**TABLE 2:** Pharmacokinetic parameters for nacubactam^*a*^

Parameter (unit)	Cohort 1 (*N* = 8)		Cohort 2 (*N* = 8)		Cohort 3 (*N* = 8)		Cohort 4 (*N* = 8)	
	Period I	Period III	Period I	Period III	Period I	Period III	Period I	Period III
*t*_max_ (h)	1.0 ± 0.0	1.0 ± 0.0	1.0 ± 0.0	1.0 ± 0.0	1.0 ± 0.0	1.0 ± 0.0	1.0 ± 0.0	1.0 ± 0.0
*C*_max_ (μg/mL)	135.9 ± 12.4	123.8 ± 10.7	129.0 ± 14.8	126.4 ± 10.4	129.3 ± 20.9	126.6 ± 12.3	118.6 ± 18.9	131.5 ± 15.5
AUC_0−t_ (μg·h/mL)	288.7 ± 22.5	280.2 ± 31.4	302.6 ± 35.7	288.2 ± 32.5	284.0 ± 42.1	292.3 ± 30.0	266.3 ± 39.9	319.4 ± 34.1
AUC_0−∞_ (μg·h/mL)	288.8 ± 22.5	280.3 ± 31.4	302.8 ± 35.7	288.3 ± 32.6	284.1 ± 42.1	292.4 ± 30.0	266.5 ± 39.7	319.5 ± 34.1
*t*_1/2_ (h)	2.5 ± 0.2	2.5 ± 0.1	2.4 ± 0.1	2.5 ± 0.1	2.5 ± 0.1	2.5 ± 0.1	2.3 ± 0.2	2.3 ± 0.1
CL (L/h)	7.0 ± 0.5	7.2 ± 0.7	6.7 ± 0.9	7.0 ± 0.8	7.2 ± 0.9	6.9 ± 0.7	7.6 ± 1.1	6.3 ± 0.7
*V*_ss_ (L)	24.9 ± 3.3	25.6 ± 3.2	23.2 ± 3.1	25.8 ± 3.1	25.9 ± 3.8	24.6 ± 2.8	25.2 ± 4.0	21.5 ± 3.4
Fe (%)	88.4 ± 3.2	87.5 ± 5.2	89.1 ± 1.8	89.3 ± 2.9	85.9 ± 11.3	90.4 ± 1.7	87.2 ± 3.5	89.2 ± 6.1
CL_*r*_ (mL/min)	102.6 ± 8.0	105.0 ± 11.7	99.5 ± 13.2	104.5 ± 13.4	103.3 ± 20.9	104.0 ± 10.2	111.3 ± 17.6	93.9 ± 11.5

^*a*^ Data are expressed as means ± standard deviations. Nacubactam was intravenously administered alone at Period I in all cohorts, whereas it was administered in combination with cefepime, aztreonam, meropenem, and piperacillin at Period III in Cohorts 1, 2, 3, and 4, respectively. *t*_max_, time to reach maximum plasma concentration; *C*_max_, maximum plasma concentration; AUC_0−t_, area under the plasma concentration-time curve from time zero to the last quantifiable time; AUC_0−∞_, area under the plasma concentration-time curve from time zero to infinity; *t*_1/2_, elimination half-life; CL, total clearance; *V*_dss_, volume of distribution at steady state; Fe, fraction (cumulative percentage) of dose excreted in urine within 24 h post-dose; CL_*r*_, renal clearance.

**TABLE 3:** Pharmacokinetic parameters for the metabolites of nacubactam^*a*^

Parameter (unit)	Cohort 1 (*n* = 8)		Cohort 2 (*n* = 8)		Cohort 3 (*n* = 8)		Cohort 4 (*n* = 8)	
	Period I	Period III	Period I	Period III	Period I	Period III	Period I	Period III
M1 (open ring analog)								
*t*_max_ (h)	1.0 ± 0.0	1.0 ± 0.0	1.0 ± 0.0	1.0 ± 0.0	1.0 ± 0.0	1.0 ± 0.0	1.0 ± 0.0	1.1 ± 0.4
*C*_max_ (μg/mL)	2.0 ± 0.1	1.3 ± 0.1	2.3 ± 0.3	1.5 ± 0.2	2.3 ± 0.3	1.8 ± 0.2	2.7 ± 0.4	2.3 ± 0.4
AUC_0−t_ (μg·h/mL)	6.9 ± 0.4	5.7 ± 0.6	8.4 ± 1.2	6.4 ± 1.1	8.0 ± 1.0	7.4 ± 0.8	8.7 ± 1.6	9.3 ± 1.5
AUC_0−∞_ (μg·h/mL)	7.1 ± 0.5	5.9 ± 0.6	8.6 ± 1.3	6.7 ± 1.2	8.2 ± 1.1	7.6 ± 0.8	8.9 ± 1.6	9.5 ± 1.5
*t*_1/2_ (h)	2.1 ± 0.1	2.2 ± 0.2	2.2 ± 0.2	2.3 ± 0.2	2.1 ± 0.2	2.2 ± 0.2	2.0 ± 0.1	2.0 ± 0.1
CL (L/h)	283.6 ± 18.9	343.4 ± 37.9	238.1 ± 43.3	308.6 ± 57.7	246.6 ± 28.9	265.2 ± 26.7	232.1 ± 45.5	216.4 ± 35.6
*V*_ss_ (L)	845.4 ± 46.8	1,073.8 ± 94.1	753.1 ± 97.2	1,016.3 ± 159.3	753.8 ± 77.1	837.1 ± 114.7	656.6 ± 118.5	629.6 ± 120.7
Fe (%)	3.4 ± 1.3	2.5 ± 0.5	3.2 ± 0.5	2.6 ± 0.5	3.4 ± 0.7	2.9 ± 0.4	2.7 ± 0.6	3.8 ± 0.6
CL_*r*_ (mL/min)	149.3 ± 54.8	132.5 ± 26.4	117.7 ± 24.1	126.0 ± 23.1	131.4 ± 27.9	122.1 ± 23.9	97.0 ± 19.0	128.9 ± 35.9
M2 (deaminated ethoxy analog)								
*t*_max_ (h)	3.3 ± 0.5	3.0 ± 0.5	3.1 ± 0.4	3.3 ± 0.5	3.1 ± 0.4	3.1 ± 0.4	2.9 ± 0.4	3.3 ± 0.5
*C*_max_ (μg/mL)	0.08 ± 0.01	0.07 ± 0.02	0.09 ± 0.02	0.07 ± 0.01	0.08 ± 0.02	0.07 ± 0.01	0.07 ± 0.01	0.09 ± 0.02
AUC_0−t_ (μg·h/mL)	0.37 ± 0.10	0.31 ± 0.09	0.49 ± 0.17	0.34 ± 0.08	0.40 ± 0.11	0.32 ± 0.09	0.31 ± 0.08	0.43 ± 0.11
AUC_0−∞_ (μg·h/mL)	0.49 ± 0.01	0.47 ± 0.08	0.62 ± 0.18	0.43 ± 0.04	0.54 ± 0.11	0.46 ± 0.07	0.43 ± 0.08	0.58 ± 0.10
*t*_1/2_ (h)	2.8 ± 0.4	3.3 ± 0.4	3.0 ± 0.5	2.9 ± 0.4	2.7 ± 0.3	3.1 ± 0.5	2.7 ± 0.4	2.2 ± 0.2
CL (L/h)	4,081 ± 74	4,313 ± 688	3,461 ± 918	4,645 ± 438	3,839 ± 903	4,441 ± 782	4,809 ± 880	3,558 ± 608
*V*_ss_ (L)	16,577 ± 2,305	20,347 ± 4,455	15,048 ± 3,926	19,238 ± 4,080	15,176 ± 4,243	20,000 ± 6,969	18,810 ± 4,441	11,376 ± 1,871
Fe (%)	0.27 ± 0.06	0.21 ± 0.04	0.31 ± 0.05	0.24 ± 0.03	0.26 ± 0.04	0.22 ± 0.03	0.19 ± 0.03	0.24 ± 0.03
CL_*r*_ (mL/min)	197.5 ± 16.3	193.6 ± 28.7	184.6 ± 36.3	200.0 ± 47.9	181.3 ± 28.9	198.4 ± 36.3	176.1 ± 30.0	158.6 ± 37.1

^*a*^ Data are expressed as means ± standard deviations. Abbreviations are explained in the footnote to Table 2.

**TABLE 4:** Pharmacokinetic parameters for cefepime, aztreonam, meropenem, and piperacillin^*a*^

Parameter (unit)	Cefepime		Aztreonam		Meropenem		Piperacillin	
	Cohort 1 (*n* = 8)		Cohort 2 (*n* = 8)		Cohort 3 (*n* = 8)		Cohort 4 (*n* = 8)	
	Period II	Period III	Period II	Period III	Period II	Period III	Period II	Period III
*t*_max_ (h)	1.0 ± 0.0	1.0 ± 0.0	1.0 ± 0.0	1.0 ± 0.0	1.0 ± 0.0	1.0 ± 0.0	1.0 ± 0.0	1.0 ± 0.0
*C*_max_ (μg/mL)	123.5 ± 8.7	124.4 ± 9.2	175.5 ± 15.6	171.6 ± 15.8	101.0 ± 12.6	107.4 ± 12.5	239.9 ± 29.8	247.5 ± 38.7
AUC_0−t_ (μg·h/mL)	311.7 ± 17.8	312.7 ± 27.8	486.9 ± 49.0	480.8 ± 43.7	183.1 ± 21.8	186.5 ± 22.5	400.1 ± 59.7	411.6 ± 75.8
AUC_0−∞_ (μg·h/mL)	311.9 ± 17.8	312.9 ± 27.8	487.2 ± 49.1	481.1 ± 43.8	183.3 ± 21.8	186.6 ± 22.5	400.3 ± 59.6	411.7 ± 75.7
*t*_1/2_ (h)	2.5 ± 0.1	2.5 ± 0.1	2.2 ± 0.1	2.3 ± 0.2	1.2 ± 0.1	1.3 ± 0.2	1.5 ± 0.5	1.5 ± 0.6
CL (L/h)	6.4 ± 0.4	6.4 ± 0.5	4.1 ± 0.4	4.2 ± 0.4	11.0 ± 1.3	10.8 ± 1.3	10.2 ± 1.6	10.0 ± 1.6
*V*_ss_ (L)	23.2 ± 1.0	23.2 ± 1.9	13.4 ± 1.1	13.6 ± 1.0	19.1 ± 3.3	20.9 ± 4.3	21.6 ± 7.1	20.7 ± 7.6
Fe (%)	93.7 ± 5.6	92.3 ± 5.0	82.4 ± 5.8	82.3 ± 3.6	59.6 ± 5.4	60.3 ± 4.8	60.5 ± 2.1	58.9 ± 3.8
CL_*r*_ (mL/min)	100.4 ± 7.2	99.0 ± 9.3	56.8 ± 5.6	57.5 ± 6.8	110.0 ± 16.6	109.3 ± 16.8	102.9 ± 17.0	97.6 ± 15.7

^*a*^ Data are expressed as means ± standard deviations. Each β-lactam antibiotic was intravenously administered alone at Period II, whereas it was administered in combination with nacubactam at Period III. Abbreviations are explained in the footnote to Table 2.

Analysis of variance confirmed the absence of drug-drug interaction in pharmacokinetics between nacubactam and each β-lactam antibiotic (Table 5). For nacubactam, the 90% confidence intervals (CIs) for the geometric least squares mean (LSM) ratios (Period III/Period I) of *C*_max_, AUC_0−t_, and AUC_0−∞_ were contained within the equivalence range of 0.80 to 1.25 in all cohorts. The 90% CIs for the geometric LSM ratios (Period III/Period II) of *C*_max_, AUC_0−t_, and AUC_0−∞_ were also contained within the range of 0.80 to 1.25 for all β-lactam antibiotics.

**TABLE 5:** Statistical comparison of pharmacokinetic parameters for nacubactam and β-lactam antibiotics between monotherapy and combination therapy (nacubactam plus each β-lactam antibiotic)

Drug	Parameter (unit)	Cohort	Ratio^*a*^ (90% CI)
Nacubactam	*C*_max_ (µg/mL)	1	0.9113 (0.8630 to 0.9623)
		2	0.9826 (0.9356 to 1.0320)
		3	0.9862 (0.9226 to 1.0542)
		4	1.1134 (1.0472 to 1.1837)
	AUC_0−t_ (µg·h/mL)	1	0.9681 (0.9188 to 1.0201)
		2	0.9530 (0.9160 to 0.9916)
		3	1.0339 (0.9886 to 1.0812)
		4	1.2050 (1.1649 to 1.2464)
	AUC_0−∞_ (µg·h/mL)	1	0.9681 (0.9189 to 1.0200)
		2	0.9531 (0.9161 to 0.9917)
		3	1.0339 (0.9887 to 1.0812)
		4	1.2043 (1.1646 to 1.2453)
Cefepime	*C*_max_ (µg/mL)	1	1.0069 (0.9851 to 1.0292)
	AUC_0−t_ (µg·h/mL)		1.0013 (0.9724 to 1.0310)
	AUC_0−∞_ (µg·h/mL)		1.0013 (0.9724 to 1.0311)
Aztreonam	*C*_max_ (µg/mL)	2	0.9777 (0.9200 to 1.0390)
	AUC_0−t_ (µg·h/mL)		0.9884 (0.9734 to 1.0036)
	AUC_0−∞_ (µg·h/mL)		0.9884 (0.9734 to 1.0036)
Meropenem	*C*_max_ (µg/mL)	3	1.0644 (1.0207 to 1.1100)
	AUC_0−t_ (µg·h/mL)		1.0180 (0.9865 to 1.0506)
	AUC_0−∞_ (µg·h/mL)		1.0181 (0.9866 to 1.0507)
Piperacillin	*C*_max_ (µg/mL)	4	1.0288 (0.9717 to 1.0892)
	AUC_0−t_ (µg·h/mL)		1.0254 (0.9767 to 1.0764)
	AUC_0−∞_ (µg·h/mL)		1.0253 (0.9767 to 1.0763)

^*a*^ Combination therapy/monotherapy. Nacubactam was administered alone at Period I, whereas cefepime, aztreonam, meropenem, or piperacillin was administered alone at Period II. Nacubactam was administered in combination with cefepime, aztreonam, meropenem, and piperacillin at Period III in Cohorts 1, 2, 3, and 4, respectively. Abbreviations are explained in the footnote to Table 2.

### Safety

No treatment emergent adverse events (TEAEs) were reported in Cohorts 1 and 4. In Cohort 2, three participants (37.5%) experienced four events (headache and feeling hot in one participant, and blood creatine phosphokinase increased in two participants). In Cohort 3, one participant (12.5%) experienced blood bilirubin increased. The increases in creatine phosphokinase (CPK) observed in two participants in Cohort 2 (496 and 567 U/L) were transient and mild, with approximately twice the upper limit of reference range (270 U/L), and were not associated with any clinical symptoms. Similarly, the elevation in total bilirubin observed in one participant in Cohort 3 (2.3 mg/dL) was mild, with approximately twice the upper limit of reference range (1.2 mg/dL), transient, and not accompanied by elevations in liver transaminases or clinical signs of hepatic dysfunction. All TEAEs were mild in severity and resolved without treatment. In addition, all TEAEs were found at the follow-up examination. Abnormal laboratory values resolved after 9 days (creatine phosphokinase increased) or 6 days (bilirubin increased) after onset. No TEAEs were considered related to the study drug. No clinically meaningful changes were observed in the laboratory test results, weight, vital signs (body temperature, blood pressure, and pulse rate), or electrocardiogram (ECG) assessments.

## DISCUSSION

In this open-label study in healthy Japanese participants, plasma concentrations of nacubactam following a single infusion of 2 g peaked at the end of the infusion followed by a rapid decrease with the mean *t*_1/2_ of 2.3 to 2.5 h. Although metabolites of nacubactam were detected in the plasma, they were formed in small amounts compared with the parent compound nacubactam. Furthermore, the analysis of urinary excretion indicated that nacubactam was excreted largely unchanged into urine with the minimal metabolic clearance. These results are consistent with those in the previous phase 1 studies (11, 12).

The coadministration of nacubactam and each β-lactam antibiotic (cefepime, aztreonam, meropenem, or piperacillin) did not affect the PK profile of nacubactam or β-lactam antibiotic. PK parameters for nacubactam were almost constant across the treatment periods and cohorts, and the PK parameters for each β-lactam antibiotic were similar when it was administered alone or coadministered with nacubactam. In addition, analysis of variance confirmed the absence of drug-drug interaction between nacubactam and each β-lactam antibiotic. For all study drugs, the 90% CIs for the geometric LSM ratios of *C*_max_, AUC_0−t_, and AUC_0−∞_ were contained within the range of 0.80 to 1.25.

β-lactam antibiotics are classified into cephalosporins, carbapenems, monobactams, and penicillins (4). Among the β-lactam antibiotics administered in this study, cefepime is a fourth-generation cephalosporin (13). Meropenem is a carbapenem with broad-spectrum activity against gram-positive and gram-negative bacteria (14). Aztreonam is a monobactam, which is stable to metallo-β-lactamase (15). Piperacillin is an extended-spectrum ureidopenicillin (16). These antibiotics are frequently partnered with β-lactam inhibitors (4, 17). Thus, our results indicate that nacubactam can be coadministered with any type of potential β-lactam partners without inducing drug-drug interaction.

In the safety assessments, nacubactam and β-lactam antibiotics were well tolerated. Although several TEAEs were reported, they were mild in severity and resolved without treatment. No clinically meaningful changes were observed in the laboratory test results, weight, vital signs, or ECG assessments. These results are consistent with those in the previous phase 1 studies (11, 12). However, the sample size in our study was limited. Future multiple-dose trials should carefully monitor laboratory values (including CPK and liver function-related markers) to evaluate potential cumulative effects. Thus, the safety of nacubactam coadministered with β-lactam antibiotic should be further assessed in the clinical studies.

In conclusion, this open-label study in healthy Japanese participants confirmed the absence of drug-drug interaction in pharmacokinetics between nacubactam and β-lactam antibiotic (cefepime, aztreonam, meropenem, or piperacillin). Nacubactam coadministered with each of these β-lactam antibiotics was well tolerated. Further studies are needed to assess the efficacy and safety of these combinations in patients with bacterial infection.

## MATERIALS AND METHODS

### Study oversight

This open-label study was conducted between 4 March 2019 and 19 April 2019 at SOUSEIKAI Sumida Hospital (Tokyo, Japan). The study complied with the principles of the Declaration of Helsinki and Good Clinical Practice guidelines. Ethical approval was obtained from the institutional review board of the study site. All participants provided written informed consent.

### Study population

Japanese healthy male volunteers aged between 20 and 39 years with the body mass index between 17.6 and 24.6 kg/m^2^ were eligible for the study, if they had no clinically abnormal findings. Key exclusion criteria included use of any medication (except for topical agents without systemic activity) within 7 days before starting the administration of the study drug or a history or current diagnosis of (i) allergic symptoms, (ii) infectious mononucleosis, or (iii) cardiac, hepatic, renal, gastrointestinal, respiratory, vascular, or hematological function disorders. Volunteers were also excluded if they had QTc interval of 450 msec or longer in the 12-lead ECG assessment. QTc interval was calculated using Fridericia’s formula.

### Study treatment

This study included four cohorts. In each cohort, participants were hospitalized to the study site from Day −1 (1 day before starting the administration of the study drug) to Day 6. Thereafter, they revisited the study site and underwent the follow-up examination on Day 12. During hospitalization, participants received a single dose of nacubactam (2 g) on Day 1, β-lactam antibiotic on Day 3, and nacubactam in combination with β-lactam antibiotic on Day 5. β-lactam antibiotics administered in Cohorts 1, 2, 3, and 4 were cefepime (2 g), aztreonam (2 g), meropenem (2 g), and piperacillin (4 g), respectively. Study drugs (nacubactam and β-lactam antibiotics) were administered as intravenous infusions over 60 min. The treatment period was divided into Periods I, II, and III, which were defined as the periods from 0 to 24 h after starting the administration of the study drugs on Days 1, 3, and 5, respectively. The planned number of participants in each cohort was 8.

### PK measurements

Blood samples for PK analysis were collected at the following time points: pre-dose and 0.5, 1, 2, 3, 4, 6, 8, 12, and 24 h after starting the administration of the study drug on Days 1, 3, and 5. Urine samples for PK analysis were also collected at the following intervals: 0–4, 4–8, 8-12, and 12–24 h after starting the administration of the study drug on Days 1, 3, and 5. The plasma and urine concentrations of nacubactam, its metabolites (M1 and M2), and β-lactam antibiotics were measured using liquid chromatography tandem mass spectrometry.

### Safety assessments

Safety assessments included monitoring of TEAEs, laboratory tests (hematology, blood chemistry, and urinalysis), and physiological tests (weight, body mass index, body temperature, blood pressure, pulse rate, and resting 12-lead ECG recording). TEAEs were coded according to the Medical Dictionary for Regulatory Activities/Japanese version 22.0.

### Statistical analysis

The safety analysis set included all participants who received nacubactam or β-lactam antibiotic at least once. The PK analysis set included all participants who received nacubactam or β-lactam antibiotic at least once and had properly measured PK data.

PK parameters were calculated according to the non-compartmental analysis using Phoenix WinNonlin (Certara USA Inc., Princeton, NJ, USA) version 6.3. In assessing the similarity of the PK profiles of nacubactam between Periods I and III, analysis of variance was used to calculate the differences in the PK parameters (*C*_max_, AUC_0−t_, and AUC_0−∞_) between these periods in each cohort, after logarithmic transformation. Back transformation provided the geometric LSM ratio (Period III/Period I) and its 90% CI for each parameter. The absence of drug-drug interaction was concluded if the 90% CIs for *C*_max_, AUC_0−t_, and AUC_0−∞_ were all contained within the range of 0.80 to 1.25. This range was based on “Guideline on drug interaction for drug development and appropriate provision of information” issued by the Japanese regulatory authority (https://www.pmda.go.jp/files/000228122.pdf). Similarity of the PK profiles of each β-lactam antibiotic between Periods II and III was also assessed in the same manner. All data were analyzed using SAS version 9.4 (SAS Institute, Cary, NC, USA).
